# Biochemical markers of contamination in fish toxicity tests

**DOI:** 10.2478/v10102-011-0015-9

**Published:** 2011-06

**Authors:** Ivana Haluzová, Helena Modrá, Jana Blahová, Marcela Havelková, Zuzana Široká, Zdeňka Svobodová

**Affiliations:** 1 Department of Veterinary Public Health and Toxicology, Faculty of Veterinary Hygiene and Ecology, University of Veterinary and Pharmaceutical Sciences Brno, Czech Republic; 2 Department of Veterinary Ecology and Environmental Protection, Faculty of Veterinary Hygiene and Ecology, University of Veterinary and Pharmaceutical Sciences Brno, Czech Republic

**Keywords:** CYP, EROD, GSH, GST, fish

## Abstract

Markers of xenobiotic metabolization (cytochrome P450, ethoxyresorufin-O-deethylase, glutathione and glutathione-S-transferase) were investigated in the liver of the common carp *Cyprinus carpio* after 28-day exposure to different pesticide formulations.

The fish exposed to herbicide Sencor 70 WG (metribuzin 700 g/kg) of 0.25 and 2.5 mg/l showed no change in cytochrome P450 and activity of ethoxyresorufin-O-deethylase when compared to control.

Successor 600 (pethoxamid 600 g/l) of 0.06; 0.22 and 0.60 mg/l did not affect either cytochrome P450 or the activity of ethoxyresorufin-O-deethylase. However, in fish exposed to Successor 600 of 0.22 and 0.60 mg/l, there was a rise in glutathione and in the activity of glutathione-S-transferase (*p*<0.05), with Spearman's correlation r = 0.23 at *p*<0.05.

Spartakus (prochloraz 450 g/l) of 0.36 and 1.08 mg/l induced cytochrome P450 and ethoxyresorufin-O-deethylase (*p*<0.05), with Spearman's correlation r=0.49 at *p*<0.01. Glutathione increased in fish exposed to 1.08 mg/l (*p*<0.05), the activity of glutathione-S-transferase rose (*p*<0.05) in all concentrations tested (0.108; 0.36 and 1.08 mg/l). Spearman's correlation between glutathione and GST was r=0.38; *p*<0.01).

The obtained data contribute to a better understanding of detoxification of the selected xenobitics in fish. Although biomarkers of the first phase of metabolization are considered to be more sensitive, our results indicate higher sensitivity of the second phase biomarkers.

**LIST OF ABBREVIATIONS**
ANC_4.5_: acid neutralization capacity
BOD_5_: biological oxygen demand
COD_Mn_: chemical oxygen demand
CYP: total cytochrome P450
EROD: ethoxyresorufin-O-deethylase
GSH: glutathione
GST: glutathione-S-transferase
m.prot.: microsoma; protein
PAHs: polycyclic aromatic hydrocarbons
PCBs: polychlorinated dibenzodionixines
PCDDs: polychlorinated dibenzodioxines
PCDFs: polychlorinated dibenzodifurans

## Introduction

Biochemical markers of contamination are important indices used in fish toxicity tests and for field monitoring of aquatic pollution. They confirm contact of the specimen with specific groups of chemical compounds and clarify their further metabolic fate. Liver (hepatopancreas) plays a key role in xenobiotic detoxication in fish. Polarity of a xenobiotic increases within two phases of metabolization through oxidation, reduction and hydrolysis reactions, subsequently the produced metabolite (or less frequently the originated compound) is conjugated with an endogenous substrate and excreted. Activity and concentrations of involved enzymatical systems and substrates can be quantified and the extent of their induction or inhibition can be compared to the control. The response is dependent on many factors, including xenobitic properties, concentration, presence of other compounds, length of exposure, water temperature, *etc*. A potential source of contamination of the aquatic environment is usage of plant protection formulations. No cases of pesticide acute poisoning have been reported in fish in the last decade, however, non-target organisms are exposed to pesticides, as many pesticide compounds are detected in surface water (Czech Hydrometeorological Institute, 2010; Modra & Svobodova, 2009).

Sencor 70 WG (metribuzin 700 g/kg) is used to control a wide range of broadleaf and grass weeds infesting potatoes, tomatoes, alfalfa, peas and other crops. Successor 600 (pethoxamid 600 g/l) is registered for use in corn, sunflowers, soyabeans, and winter rape. The formulation is intended to control annual poaceous and dicotyledonous weeds. Spartakus (prochloraz 450 g/l) is used to reduce fungal diseases in barley, rye and ornamentals.

The aim of this work was to assess selected biochemical markers of contamination in fish through toxicity tests. Sublethal effects of plant protection formulations on juvenile common carp *Cyprinus carpio* were evaluated under subchronic conditions.

## Materials and methods

Three experiments with commercial plant protection formulations were conducted on juvenile common carp *Cyprinus carpio*. The exposure lasted for 28 days. The active ingredients were metribuzin (700 g/kg of Sencor 70 WG; Bayer CropScience AG - Germany) belonging to triazinone family, pethoxamid (600 g/l of Successor 600; Stähler International GmbH & Co. - Germany) belonging to chloroacetamides and an imidazole compound prochloraz (450 g/l of Spartakus; BASF SE - Germany).

The fish were obtained from commercial fish farms and acclimatized to laboratory conditions for 14 days. They were supplied twice a day with commercial feed at a total rate of 1.5% of body weight. A photoperiod of 12/12 hr was used. The tests were conducted in a semistatic system (100 l fishtanks, exchange of the test solutions every 48 hr) and flow-through system (200 l fishtanks, bath exchange twice during 24 hr). Details of the test conditions are shown in Table 1. All concentrations and controls were performed in duplicate, the physico-chemical parameters of the water used in the tanks were: ANC_4.5_=4.20 mmol/l, COD_Mn_=2.80 mg/l, BOD_5_=0.72 mg/l, NH_3_+NH_4_^+^=not detected, NO_3_^−^=23.48, NO_2_^−^=not detected, Cl^−^=18.11 mg/l.

**Table 1:** Conditions of 28-day tests of plant protection formulations on common carp *Cyprinus carpio.*

formulation (a.i.) chemical name of the a.i.	test conc. of the formulation [mg/l] s. per conc./ f. per aquarium	test system	initial fish weight [g]	test bath characteristics		
				t [°C]	pH	O_2_ [%]
**Sencor 70 WG (metribuzin 700 g/kg)**	0; 0.25; 2.5	semistatic	34-44	21±2	8.1-8.4	> 60
4-amino-6-(1,1-dimethylethyl)-3-methylthio-1,2,4-triazin-5(4H)-on	n=19-23/12					
**Successor 600 (pethoxamid 600 g/l)**	0; 0.06; 0.22; 0.60	semistatic	75±15.0	21±1.5	7.5-8.2	> 60
2-chloro-*N*-(2-ethoxyethyl)-*N*(2-methyl-1-phenylprop-1-enyl)acetamid	n=23-24/12					
**Spartakus (prochloraz 450 g/l)**	0; 0.108; 0.36; 1.08	flow-through	218±64.5	19-22	7.1-7.9	> 60
*N*-propyl-*N*-[2-(2,4,6-trichlorophenoxy)ethyl]imidazol-1-carboxamid	n=30/15					

a.i. - active ingredient, s. - samples, f. - fish, conc. - concentration

After the stipulated exposure period, blood was sampled, the fish were euthanised and their tissues were dissected. Further, only analyses of xenobiotic metabolizing systems are reported and their results discussed in this paper. Sa mples of liver (hepatopancreas) were stored at -85 °C until they were further processed. The thawed samples were homogenized in buffer (pH 7.4), centrifuged (10,000 g; 20 min; 4 °C) and the supernatant was re-centrifuged (100,000 g; 1 h at 4 °C). The final supernatant was drained and the pellet was washed and resuspended in the buffer (pH 7.4). Each suspension was put into an Eppendorf tube and stored at -85 °C until enzymatic assays. The concentration of total cytochrome P450 was quantified spectrophotometrically at 400-490 nm, on the basis of the difference between absorbance readings at 450 and 490 nm. Activity of EROD was determined spectrofluorimetrically (Chang and Waxman, 1998; Nilsen *et al*., 1998; Rutten *et al*., 1992) for excitation/emission wavelenghts setting 535/585 nm. Both methods are described in detail by Siroka *et al*. (2005). Concentrations of microsomal protein were measured according to Lowry *et al*. (1951) before performing the assays.

Prior to determination of glutathione content, activity of glutathione-S-transferase and protein concentration in liver samples, they were individually extracted with a phosphate buffer (pH 7.2), homogenized and centrifuged (10,500 g; 20 min; 4 °C). The activity of GST was investigated in supernatants spectrophotometrically by the method of Habig *et al*. (1974). Glutathione content was measured spectrophotometrically at 414 nm using Ellman's method (1959). Concentrations of GSH (nmol/mg protein) were calculated according to a standard calibration. The protein concentrations were quantified with the Bicinchoninic Acid Protein Assay Kit (Sigma-Aldrich) using bovine serum albumin as a standard.

Due to insufficient amount of samples from fish exposed to Sencor 70 WG, only total CYP and activity of EROD were assessed in them.

The obtained data were processed with Unistat 5.1 Software and evaluated for analysis of variance ANOVA - Tukey test. Spearman's rank correlation was used to test relation between CYP and EROD and between GSH and GST. Results are expressed as mean±SD.

## Results

### CYP and EROD

There was no statistically significant difference in CYP and EROD in fish exposed to Sencor 70 WG when compared to the control group (control, the formulation of 0.25 and 2.5 mg/l - CYP: 0.04±0.03; 0.04±0.04; 0.04±0.03 nmol/mg m. prot. EROD: 8.18±7.78; 4.79±4.50; 2.93±3.61 pmol/min/mg m. prot.).

CYP and EROD were not significantly influenced by the test concentrations of Successor 600 (control; the formulation of 0.06; 0.22 and 0.60 mg/l - CYP: 0.17±0.06; 0.13±0.06; 0.18±0.08; 0.20±0.07 nmol/mg m. prot. EROD: 19.78±11.99; 22.52±10.91; 17.72±11.37; 22.82±9.27 pmol/min/mg m. prot.).

**Figure 1:**
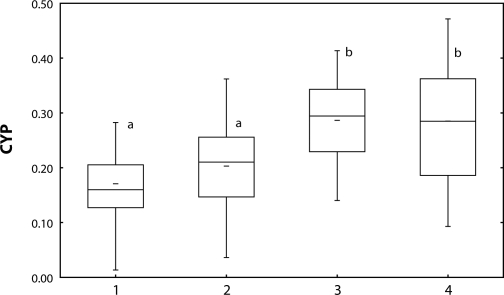
Concentration of CYP [nmol/mg m. prot.] in carp liver after 28-day exposure to Spartakus. 1=control; 2–4=fish exposed to Spartakus (2=0.108 mg/l; 3=0.36 mg/l; 4=1.08 mg/l) a, b=different alphabetic letters differ significantly (*p*<0.05) Median=Middle line of the box; Lower (Upper) Quartile=Bottom (Top) line of the box; Lower (Upper) whisker=Lower (Upper) adjacent value.

**Figure 2:**
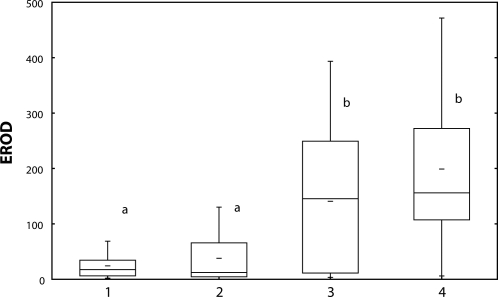
EROD activity [pmol/min/mg m. prot.] in carp liver after 28-day exposure to Spartakus. 1=control; 2–4=fish exposed to Spartakus (2=0.108 mg/l; 3=0.36 mg/l; 4=1.08 mg/l) a, b=different alphabetic letters differ significantly (*p*<0.05) Median=Middle line of the box; Lower (Upper) Quartile=Bottom (Top) line of the box; Lower (Upper) whisker=Lower (Upper) adjacent value.

Total CYP and activity of EROD were induced by Spartakus of 0.36 mg/l and 1.08 mg/l (Figures 1 and 2). Spearman's correlation between CYP and EROD was r=0.49 (*p*<0.01).

### GSH and GST

**Figure 3:**
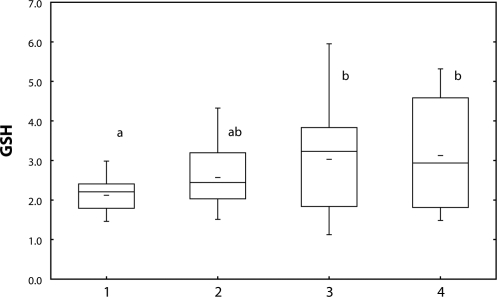
Glutathione [nmol/mg protein] in carp liver after 28-day exposure to Successor. 1=control; 2–4=fish exposed to Successor 600 (2=0.06 mg/l; 3=0.22 mg/l; 4=0.60 mg/l) a, b=different alphabetic letters differ significantly (*p*<0.05) Median=Middle line of the box; Lower (Upper) Quartile=Bottom (Top) line of the box; Lower (Upper) whisker=Lower (Upper) adjacent value.

**Figure 4:**
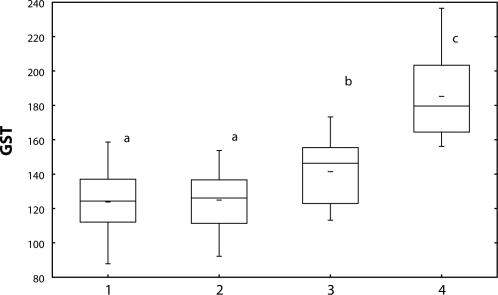
Glutathione-S-transferase activity [nmol/min/mg protein] in carp liver after 28-day exposure to Successor 600. 1=control; 2–4=fish exposed to Successor 600 (2=0.06 mg/l; 3=0.22 mg/l; 4=0.60 mg/l) a, b, c=different alphabetic letters differ significantly (p<0.05) Median=Middle line of the box; Lower (Upper) Quartile=Bottom (Top) line of the box; Lower (Upper) whisker=Lower (Upper) adjacent value.

The content of glutathione (Figure 3) as well as activity of GST (Figure 4) increased in fish treated with Successor 600 of 0.22 mg/l and 0.60 mg/l (*p*<0.05), with Spearman's correlation r=0.23 (*p*<0.05).

**Figure 5:**
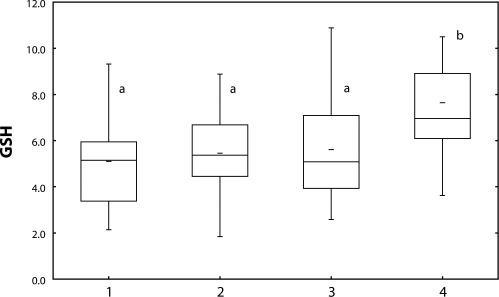
Glutathione [nmol/mg protein] in carp liver after 28-day exposure to Spartakus. 1=control; 2–4=fish exposed to Spartakus (2=0.108 mg/l; 3=0.36 mg/l; 4=1.08 mg/l) a, b=different alphabetic letters differ significantly (*p*<0.05) Median=Middle line of the box; Lower (Upper) Quartile=Bottom (Top) line of the box; Lower (Upper) whisker=Lower (Upper) adjacent value.

**Figure 6:**
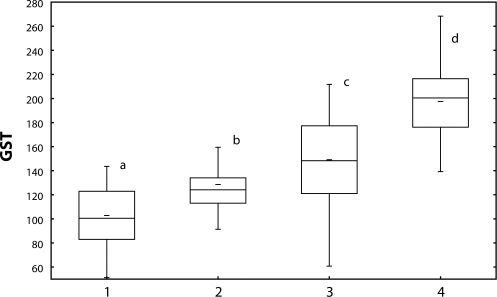
Glutathione-S-transferase activity [nmol/min/mg protein] in carp liver after 28-day exposure to Spartakus. 1=control; 2–4=fish exposed to Spartakus (2=0.108 mg/l; 3=0.36 mg/l; 4=1.08 mg/l) a, b, c, d=different alphabetic letters differ significantly (*p*<0.05) Median=Middle line of the box; Lower (Upper) Quartile=Bottom (Top) line of the box; Lower (Upper) whisker=Lower (Upper) adjacent value.

Spartakus of 1.080 mg/l enhanced glutathione content (Figure 5) in fish liver (*p*<0.05), GST activity (Figure 6) was induced by all concentrations tested (*p*<0.05). Spearman's correlation between the indices was r=0.38 (*p*<0.01).

## Discussion

Biochemical markers of the first (CYP, EROD) and second phase of detoxification (GSH, GST) are significant indicators of pollutant exposure in fish. Biotransformation of a wide range of xenobiotics is performed by CYP1A subfamily, whose catalytic activity is expressed as activity of EROD. Well-established inducers of CYP1A and EROD are agonists of Ah receptor - particularly organic contaminants belonging to PCBs, PAHs, PCDDs and PCDFs. However, induction by pesticide compounds has also been reported (Mikula *et al*., 2009; Dong *et al*., 2009). The lack of response of CYP to exposure by Sencor 70 WG and Successor 600 could be caused by either induction of specific isoenzymes and concurrent inhibition of others, resulting in a non-significant effect on total cytochrome P450, although isoenzyme levels were altered (van der Oost *et al*., 2003), or cytochrome P450 was not involved in detoxification of the compounds tested. The mechanism of metribuzin metabolism in fish has not been studied previously. However, the importance of cytochrome P450 for metribuzin detoxification in mice was demonstrated by Bleeke *et al*. (1985).

High concentration of pollutants and the presence of contaminants specifically inhibiting cytochrome P450 also prevent the induction of CYP1A. Typical agonists of Ah receptor are of a planar, aromatic and hydrophobic nature. Planar conformation of prochloraz (imidazole family) is unlikely. However, it is possible that imidazoles are converted intracellularly into planar metabolites which then bind as ligands to the Ah receptor or activate Ah receptor via alternative intracellular signalling mechanisms (Babin *et al*., 2005).

In the second phase of metabolization, hydrophilicity of metabolites (or parent compounds) is futher enhanced by addition of a more polar group, facilitating their excretion. The major pathway for nucleophilic compounds is conjugation with glucuronic acid by UDP-glucuronyl transferase, while electrophilic compounds are conjugated with glutathione (George, 1994). The reaction is primarily catalyzed by glutathione-S-transferase. Although the reaction is generally detoxication, increased bioreactivity of several intermediates has been assumed (Balendiran *et al*., 2004). The enzyme is also involved in the transport of endogenous hydrophobic substances such as steroids, bilirubin, heme and bile salts and consequently in the synthesis of prostaglandins and leucotriens (Blanchette *et al*., 2007). Se-independent glutathione-peroxidase activity of GST seems to play an important role in protection against lipid peroxidation (Yang *et al*., 2001). Glutathione is the major cell antioxidant. It serves as a substrate for the antioxidant enzymes and protects cells also via non-enzymatic scavenging of free radicals. Glutathione also binds metals before induced synthesis of metallothionines reaches effective levels (Kovarova & Svobodova, 2009). In laboratory tests and in field monitoring, both induction and inhibition of the second phase biomarkers have been reported (van der Oost *et al*., 2003).

The biomarkers of the second phase of metabolization are generally considered less sensitive than the first phase systems. However, the results of our tests are not in agreement with this statement. We revealed induction of both EROD and CYP as well as glutathione and GST (Spartakus - prochloraz) or the first phase parameters did not response to exposure (Sencor 70 WG - metribuzin, Successor 600 - pethoxamid) although the glutathione and GST indicated the exposure (Successor 600). Glutathione conjugation has been suggested as the major metabolic pathway of pethoxamid in animals, plants and soil (Kato *et al*., 2001). Debrauwer *et al*. (2001) and Cravedi *et al*. (2001) reported glucuronide conjugates of prochloraz in rainbow trout *Oncorhynchus mykiss*. An increase in GST activity in *Gasterosteus aculeatus* after exposure to prochloraz was observed by Sanchez *et al*. (2008).

The results of toxicity tests lead to identification of xenobiotics as inducers or inhibitors of particular detoxification systems. Thus, interpretation of biomonitoring studies can be more easily associated with findings of *e.g.* water or sediment analyses. However, the potential counteractive effect of mixtures of pollutants should be also taken into consideration.
